# ﻿First records of *Oxychilusalliarius* and *O.cellarius* (Gastropoda, Stylommatophora, Oxychilidae) in Mexico: mtDNA identification and potential distributions

**DOI:** 10.3897/zookeys.1224.129618

**Published:** 2025-01-28

**Authors:** Ali Gabrielle Trujillo-Díaz, Victoria Araiza-Gómez, Jazmín García-Román, José Luis Hernández-Domínguez, Gerardo Zúñiga, Edna Naranjo-García

**Affiliations:** 1 Laboratorio de Variación Biológica y Evolución, Escuela Nacional de Ciencias Biológicas, Instituto Politécnico Nacional, Prolongación de Carpio y Plan de Ayala, Col. Santo Tomas, Alc. Miguel Hidalgo, Ciudad de México CP 11340, Mеxico Escuela Nacional de Ciencias Biológicas, Instituto Politécnico Nacional México City Mexico; 2 Centro de Investigación en Alimentación y Desarrollo, Unidad Mazatlán, Av. Sábalo-Cerritos S/N, Sinaloa, C.P. 82100, Mexico Centro de Investigación en Alimentación y Desarrollo Sinaloa Mexico; 3 Universidad Autónoma de Nuevo León, Facultad de Ciencias Forestales, Kilómetro 145, Nacional 85, 67700 Linares, N.L, Mexico Universidad Autónoma de Nuevo León Nuevo León Mexico; 4 Agencia digital de Innovación Pública, Dirección Ejecutiva de Inteligencia de Datos, Plaza de las Vizcaínas No.30, Col. Centro, Alc. Cuauhtémoc, Ciudad de México CP 06080, Mexico Agencia digital de Innovación Pública, Dirección Ejecutiva de Inteligencia de Datos México City Mexico; 5 Departamento de Zoología, Instituto de Biología, Universidad Nacional Autónoma de México, Apartado postal 70-153, 04510 México, D. F., Mexico Universidad Nacional Autónoma de México México City Mexico

**Keywords:** COI, distribution, introduced species, land snail, Mollusca, taxonomy

## Abstract

This paper reports the first Mexican records of *Oxychilusalliarius* (Puebla, State of Mexico, Mexico City) and *O.cellarius* (Mexico City), and expands the Mexican distribution of *O.draparnaudi* to Querétaro, Tlaxcala, and State of Mexico. These three introduced land snail species were identified by combining their genital anatomy and mitochondrial COI DNA sequence data. A two-dimensional geometric morphometric analysis of shell shape variation based on both apertural and apical views showed that there were no significant conchological differences between the three species except, to some degree, size. Using locality data of newly collected specimens, information from previous studies, and data retrieved from GBIF and iNaturalist, an analysis of the potential distributions of *Oxychilus* species in Mexico was conducted with an R implementation of Maxent. This showed that *Oxychilus* tends to occupy principally the Southern Highlands and the Transmexican Volcanic Belt Province.

## ﻿Introduction

Biological invasions modify native diversity, ecosystem functioning and species interactions (Lankau 2013). Non-native species invasions often negatively affect community structure, although positive effects can sometimes occur (Geerts and Pauw 2009; Simberloff et al. 2013). These outcomes depend on whether species compete for single or multiple factors (Case 1990; Godoy 2019). The detrimental impacts of introduced species on native biodiversity have been extensively discussed worldwide (McNeely 2001; Meyer and Cowie 2010). Research has focused on identifying common patterns in invasion events, examining the intrinsic characteristics of invaders, the vulnerability of natural communities to invasion, and the relationship between invader distribution and environmental factors (Kappes et al. 2009; Stoll et al. 2012). However, studies addressing the impacts and distribution of introduced land snails in Mexico remain scarce (Naranjo-García and Castillo-Rodríguez, 2017). Land snails are a highly diverse group of animals that have received relatively little attention in Mexico, despite their important roles in ecosystem functioning (Correa et al. 2012). One widely distributed and increasingly expanding land snail genus is *Oxychilus* Fitzinger, 1833 (Curry et al. 2016; Ali and Robinson 2020). The genus *Oxychilus* (family Oxychilidae Hesse, 1927) includes species that are usually found in humid habitats, under rocks, among moss, and near water bodies. The genus comprises 103 species (MolluscaBase 2021), characterized by thin shells that are either depressed, discoidal, or slightly elevated with a rounded periphery. The shells have an umbilicus and a semilunar aperture, and their coloration ranges from yellowish to brown or even whitish (Correa-Sandoval et al. 2017). However, species identification and generic assignment are challenging because of the extreme conchological similarities between both species and genera (Falkner 2008; Bronne and Delcourt 2022).

*Oxychiluscellarius* (O.F. Müller 1774) and *O.draparnaudi* (H. Beck, 1837) are native in Europe and have been introduced in many other parts of the world. As such, *O.draparnaudi* has also been recorded in the Canary Islands, the Azores, and numerous parts of North and South America (Virgillito and Miquel 2013), South Africa and New Zealand (Brook 1999; MacDonald et al. 2004), among others. In Mexico, Naranjo-García and Castillo-Rodríguez (2017) reported *O.draparnaudi* from Coyoacán, Mixcoac, Bosque de Chapultepec, Bosque de Tlalpan, and Pedregal de San Ángel in Mexico City (CDMX). *Oxychiluscellarius*, in turn, has been reported from Asia Minor, North Africa, the United States, and Chile, including Juan Fernández Islands and Santiago (Stuardo and Vega 1985). Valdovinos (1999) situated its distribution between latitudes 30°S and 40°S. *Oxychilusalliarius* (J.S. Miller, 1822) is a third *Oxychilus* species that has been recorded outside its native European range. It occurs in Colombia, North America, Greenland, St. Helena, South Africa, Sri Lanka, Australia, New Zealand, Hawaii, and the Juan Fernández Islands (Stuardo and Vega 1985). This species selectively preys on other snails smaller than 3 mm (Frömming 1954; Meyer 2005) and Stone and Scott (1985) claimed that it is an invasive species that occurs in higher densities than other snail species, by which it contributes to the decline of these other species (Meyer 2005).

In this paper we report for the first time the presence in Mexico of *O.cellarius* and *O.alliarius* using anatomical and mtDNA data. Additionally, we assess the potential future spread of these invasive species in new habitats by analyzing their distribution patterns in Mexico through habitat suitability modeling.

## ﻿Materials and methods

We used *Oxychilus* samples deposited in the National Collection of Mollusks (**CNMO**) at the Institute of Biology (**IB-UNAM**), specimens stored in the laboratory of Biological Variation and Evolution at the National School of Biological Sciences (**ENCB-IPN**), and recent collections made from July to November 2021 and in November 2022 (Suppl. material 1: table S1). The collected specimens were relaxed by immersing them in a container filled with boiled cold water. They were preserved in 95% ethanol for anatomical and DNA analysis.

To recognize the conchological attributes of the genus *Oxychilus*, we employed specialized literature (Giusti and Manganelli 1997, 2002; Sysoev and Schileyko 2009; Thompson 2011). Characteristic attributes such as the number of whorls, color, shape, and ornamentation were compared with descriptions in the literature. Furthermore, shell diameter, shell height, and umbilicus diameter were measured using a caliper. Snails were dissected (15 *O.draparnaudi*, two *O.alliarius*, and two *O.cellarius*) under a Nikon SMZ800 stereomicroscope and diagnostic anatomical structures were photographed.

### ﻿mtDNA identification

DNA was extracted from a fragment of the foot using the DNeasy Tissue kit according to the manufacturer’s specifications (QIAGEN, California, USA). A 600-bp fragment of the mitochondrial cytochrome oxidase subunit 1 (COI) gene was amplified by PCR using the universal primers LCO1490 and HCO2198 (Folmer et al. 1994). PCR amplifications were performed with a thermocycler (Techne TC-5000) on 25 μl reaction volume containing 2.5 μl of 10X PCR buffer, 2.5 mM MgCl_2_ 10 mM dNTPs, 13 pmol of primers, 1U Taq DNA polymerase (Invitrogen) and 2 μl of template DNA. Cycling conditions were an initial 5 min denaturation step at 94 °C, followed by 35 cycles: 94 °C for 30 s, 50 °C for 30 s, and 72 °C for 45 s; and a final step at 72 °C for 7 min. The PCR products were purified using the GFXTM PCR DNA and GelBand Purification Kit (GE Healthcare, Buckinghamshire, UK) and sequenced by Macrogen, Korea.

The nucleotide sequences of the COI fragment were compared with sequences deposited in GenBank (http://www.ncbi.nlm.nih.gov) using BLASTn. Sequences were aligned and manually edited using CHROMAS v. 2.33 (http://www.technelysium.com.au/chromas.html) and SEAVIEW v. 4.0 (Gouy et al. 2010) software programs. After editing the sequences, a 450-bp fragment was used for phylogenetic analysis.

Phylogenetic analysis was performed by Bayesian inference with BEAST v. 2.7.3 (Drummond and Rambaut 2007) under the HKY substitution model. Tree runs were performed independently with four Markov chains that went for 10,000,000 generations, resampling every 10,000 states and a Burn-in of 10%. The new sequences were deposited in GenBank under the accession numbers PP431570, PP431571, PP431572, PP431573, PP431574, PP658222, PP658223, PP658224, and PP942456.

### ﻿Geometric morphometrics analysis

We examined shell shape variation with a two-dimensional geometric morphometric analysis, under the hypothesis that *O.draparnaudi*, *O.cellarius*, and *O.alliarius* represent three shell morphotypes that were defined by their genital anatomy. Shell shape variation was analyzed both in apertural and apical views, quantified through landmarks and semi-landmarks placed on photographs of the individual shells (Bookstein 1982; Zelditch et al. 2004). Shell images were captured using a camera attached to a stereomicroscope Nikon SMZ800, ensuring that the suture of the spire (in apical view) or the shell aperture (in apertural view) were observed in the same plane in all shells of either series.

**Figure 1.**
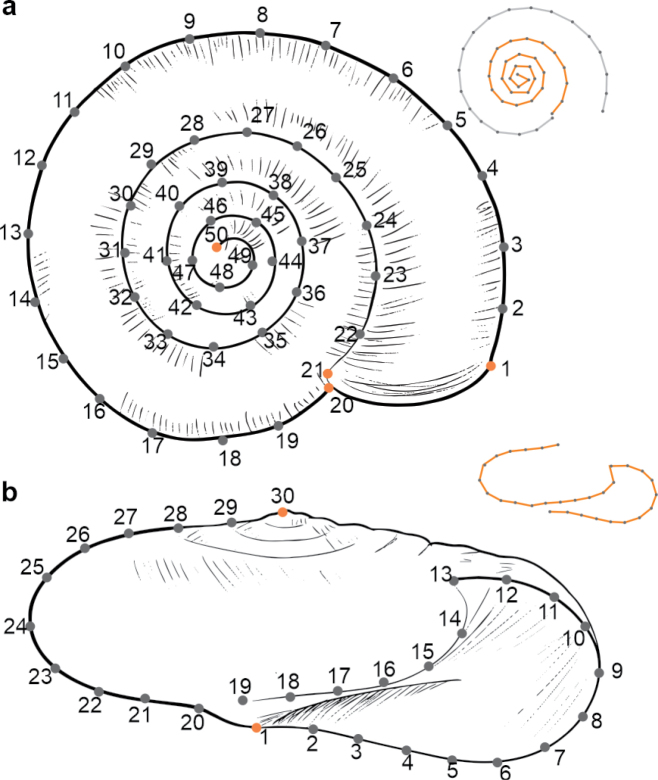
Landmarsks *sensu stricto* (orange dots) and semi-landmarks placed on *Oxychilus* shells **a** apical view **b** apertural view.

**Figure 2.**
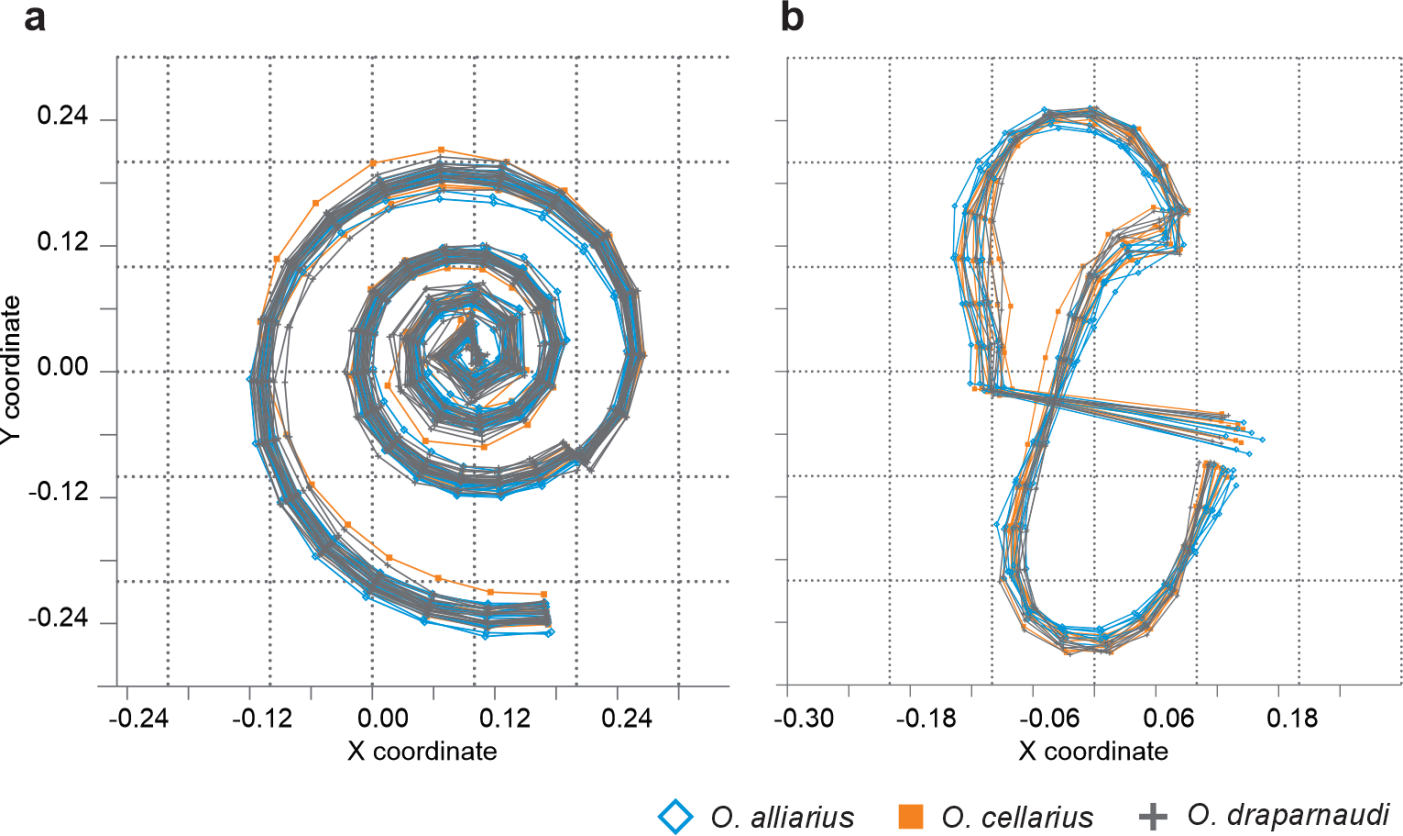
Superimposition of landmark coordinates for the three morphotypes hypothesis **a** apical view **b** apertural view.

A total of 22 apertural view photographs (eight *O.cellarius*, seven *O.alliarius*, and seven *O.draparnaudi*) and 39 apical view photographs (five *O.cellarius*, 17 *O.alliarius*, and 17 *O.draparnaudi*) were obtained. For the apical view, 50 semi-landmarks were used, while for the apertural view, 30 semi-landmarks were used (Fig. 1a, b). The x, y coordinates of the landmarks and semi-landmarks were digitized in the program TPSDIG2 v. 2.32 (Rohlf 2004), using the ‘draw background curves’ function, placing equidistant semi-landmarks along curves. In the apical view, two curves were used, one corresponding to the outer curvature of the shell starting at the distal point of the shell aperture and ending at the proximal point of the same aperture, and the other corresponding to the spire sutures (Fig. 2a); for the apertural view, only one curve that runs along the entire shell aperture, passing through the body whorl to the embryonic spire, was used (Fig. 2b).

To minimize the tangential variation of the semi-landmarks, a coordinate adjustment was performed using the SEMILAND6 program from the IMP suite (Sheets 2014). To superimpose the specimen coordinates and extract relevant shape data for comparison, a Generalized Procrustes Analysis (GPA) was performed using the SEMILAND6 program from the IMP suite, in which all sets of landmarks were translated around a common origin to remove the effect of position, scale, and orientation (Masonick and Weirauch 2020).

Shell shape variation was evaluated using a Principal Coordinates Analysis (PCoA) in the PAST program v. 4.08 (Hammer and Harper 2006), based on the covariance matrix of individuals, obtaining a scatterplot with the two components that accounted for the highest percentage of variation. To represent the overall shape modification of each component, thin-plate spline deformation grids were calculated from the variability of the average shape along each PCoA.

### ﻿Distribution data

Species distribution data were obtained from (1) the information on the labels of the specimens deposited in the IB-UNAMCNMO, (2) the Global Biodiversity Information Facility database (Hammer and Harper 2006), and (3) iNaturalistMX (https://mexico.inaturalist.org/). The citizen observations in iNaturalistMX were reviewed by VAG, considering only those that matched the characteristics of the genus. Blurry photos and erroneous identifications (two records involved *Succinea* sp., two records to family Polygyridae, one to family Euconulidae, and one was an insect larva) were removed. These corrections were documented on the iNaturalistMX observation platform. With these records, a data table was built with the following attributes: 1) latitude, 2) longitude, 3) source from which the record was obtained and 4) locality (Suppl. material 1: table S2). Records with incomplete locality data that did not allow locating the collection site were excluded from the analysis.

### ﻿Potential distribution

**Figure 3.**
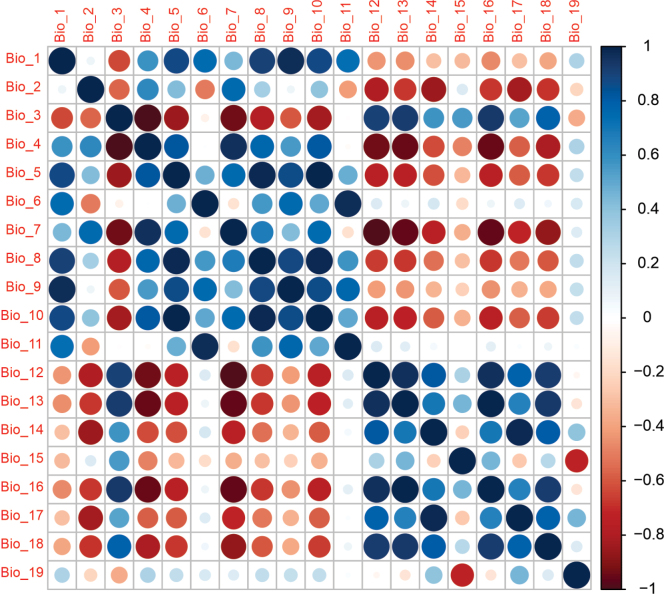
Spearman pairwise correlation coefficients between predictive variables. Variables selected for the final model were not highly correlated (r < 0.7).

The model was created utilizing an R implementation (R Core Team 2024) of Maxent from the Maxnet library (Phillips 2021) and SDMtune (Vignali et al. 2022) for the training and evaluation of distribution models. Maxent employs the principle of maximum entropy to estimate the probability distribution of a species using presence and absence data. The species habitat modeling was done with 142 *Oxychilus* spp. occurrence locations in Mexico: 26 from our collections and the CNMO records, one from GBIF (a specimen in the Carnegie Museum of Natural History) (GBIFa 2022; GBIFb 2023) and 115 from iNaturalistMX. We employed 19 bioclimatic variables downloaded from the WorldClim v. 2.1 (http://worldclim.org/version2) with a spatial resolution of 30 s. The model establishes a statistical connection between the environmental factors found at the locations where a species is observed and data indicating the species presence. It is commonly used for habitat modeling, even when there is limited information about the species occurrences (Phillips et al. 2006). The model randomly selects background points across the study area, representing locations where the species is considered to be either truly absent or likely absent (pseudo-absent). The relative significance of the environmental variable was calculated using Jackknife analysis inbuilt in the model. Using jackknife and Spearman correlation analysis we included the variables which were not highly correlated (r < 0.7) (Fig. 3). The model was evaluated with the help of AUC (Area under curve) of the ROC (Receiver operating characteristics) plot and true skill statistics (TSS) (Allouche et al. 2006). AUC in percentages measures model performance and varies from random to perfect discrimination (Swets 1988; Fawcett 2006).

## ﻿Results

### ﻿Collections and morphological identifications

We analyzed 50 specimens (Suppl. material 1: table S1) and 74 shells from four states of Mexico in two biogeographical provinces (Fig. 12). The live snails were found under logs and damp wood in (1) Tlaxcala (Atlihuetzia) where they lived in sympatry with *Deroceraslaeve* (O.F. Müller), 1774, *D.reticulatum* (O.F. Müller 1774), *Arionintermedius* Normand, 1852, and *Boettgerillapallens* (Simroth, 1912), (2) Querétaro where they were found in sympatry with *D.laeve*, (3) CDMX (Bosque de Tlalpan) where they co-occurred with *D.laeve* and *B.pallens*, and (4) Puebla (Teopancingo) where they were found in sympatry with *B.pallens* and *Pallifera* sp. The newly collected specimens were deposited at the **CNMO** (Suppl. material 1: table S1).

### ﻿Systematics

**Superfamily Gastrodontoidea Tryon, 1866**

**Family Oxychilidae Hesse, 1927**

**Subfamily Oxychilinae Hesse, 1927**

#### ﻿Genus Oxychilus

Taxon classificationAnimaliaStylommatophoraOxychilidae

Fitzinger, 1833

A3C973EA-CE8E-5E99-BDF0-230DBFA90A7E

##### Type species.

*Helixcellaria* O.F. Müller, 1774 (type designation: Herrmannsen 1847).

#### ﻿ Oxychilus draparnaudi

Taxon classificationAnimaliaStylommatophoraOxychilidae

(H. Beck, 1837)

7D49B29A-384A-5E86-9A58-32648A9D87CC

Fig. 4

##### Worldwide distribution.

Originally described from France, probably in the Montpellier area (Giusti and Manganelli 1997). Its native range includes western Europe and the western Mediterranean region (Barker 1999). It has spread to other parts of the world, including North America (Forsyth 2004) Russia, North and South Africa, Asia, Australia, and New Zealand (Barker 1999), Madeira (Seddon 2008), and Argentina (Virgillito and Miquel 2013).

##### Distribution in Mexico.

Querétaro (Cadereyta de Montes), Tlaxcala (Atlihuetzia), State of Mexico (Tlalnepantla), CDMX (Álvaro Obregón, Benito Juárez, Tlalpan). According to Morrone (2019), localities belong to the Sierra Madre Oriental and Transmexican Volcanic Belt Province.

##### Diagnostic features.

**Figure 4.**
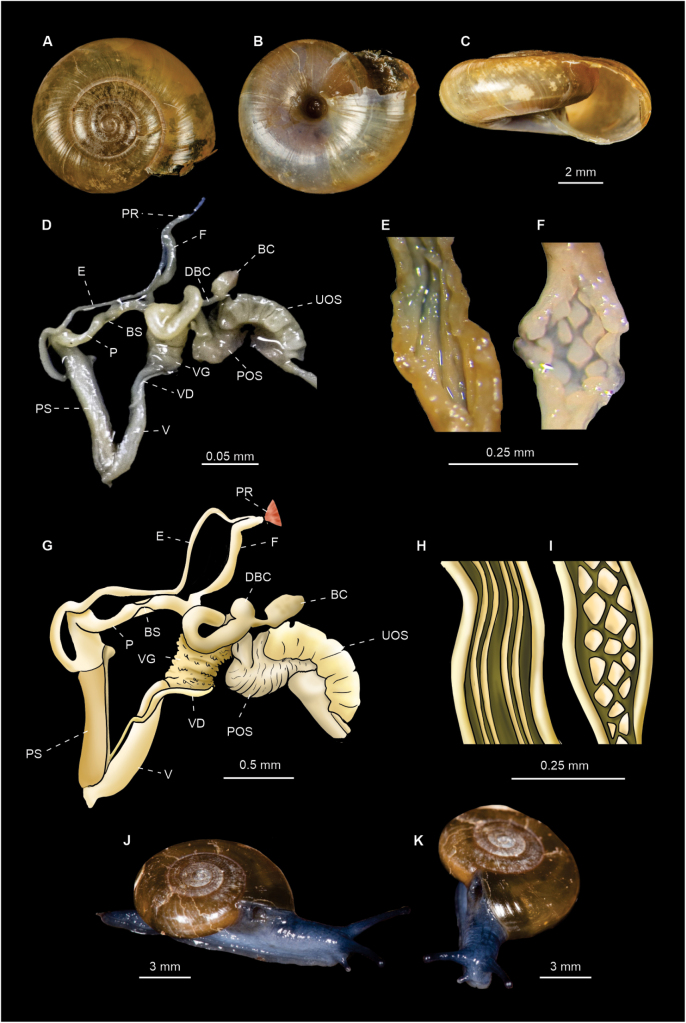
*Oxychilusdraparnaudi* (CNMO 8470 Cadereyta, Querétaro). **A–C** shell in apical, ventral, and apertural view **D, G** genitalia of *O.draparnaudi***F, I** internal ornamentation of the proximal penis **E, H** internal ornamentation of distal penis **J, K** live specimens. Abbreviations: BS bottleneck region, BC bursa copulatrix, DBC bursa duct, E epiphalus, F flagellum, P penis, POS prostatic portion of ovospermiduct, PR penial retractor, PS penial sheath, UOS uterine portion of ovospermiduct, V vagina, VD vas deferens, VG vaginal gland.

15 specimens were dissected. Anatomically, all of them displayed the genital features typical of *O.draparnaudi* as described by Giusti and Manganelli (1997): a penis with a very slender proximal portion and a wider distal portion, both separated by a “bottle-neck” (= BS, i.e., a twisted duct or constriction) covered by a thin translucent sheath (Fig. 4D, G). Internally the proximal penis shows prominent papillae (Fig. 4F, I), while the distal penis shows four to five thin longitudinal internal folds (Fig. 4E, H).

***Shell*** discoidal (Fig. 4B, C) with a depressed spire (Burch 1962), thin, yellowish, shiny; shell surface generally smooth with fine growth lines most evident near the suture and few very fine spiral lines (Fig. 4A). Shell whorls: 5 ½ to 6. Umbilicus moderately wide, 1⁄6 of maximum shell diameter. Shell diameter 9–13 mm, shell height 3–6 mm (Fig. 7). Aperture width: 3.00–4.95 mm.

***Radula*** (*n* = 5) composed of 30 rows with ~ 25 teeth/row (Fig. 8A). Radular formula: C/3 + 2–3 L/3 + 0–1 LM/2 + 9–12 M/1.

#### ﻿ Oxychilus alliarius

Taxon classificationAnimaliaStylommatophoraOxychilidae

(J.S. Miller, 1822)

D6758219-EAF1-544C-B2A5-B6D143D77CAD

Fig. 5

##### Worldwide distribution.

Originally described from the “Environs of Bristol”, England (Miller 1822). Its native distribution includes Iceland, Greenland (Roth and Sadeghian 2003), and Central and Western Europe (Horáčková and Juřičková 2009). It has been reported as introduced in Austria, Belgium, Denmark, Estonia, Finland, France, Germany, Great Britain, Greece, Czech Republic, Spain, and Portugal (Horáčková and Juřičková 2009; Borges et al. 2010; De Oliveira and Altonaga 2010; IUCN Red List 2017), and spread to Hawaii (Cowie 1997), Tasmania (Kershaw 1991), South Atlantic (Preece 2001), North America (Forsyth 2004), Colorado, the Pacific coast states (Roth and Sadeghian 2003), South America (Hausdorf 2002), Chile (Cádiz et al. 2013), Sri Lanka (Naggs et al. 2003), Madeira (Seddon 2008), and the Philippines (Tañan and Sumaya 2024).

##### Distribution in Mexico.

Puebla (Teopancingo), Transmexican Volcanic Belt Province.

##### Diagnostic features.

**Figure 5.**
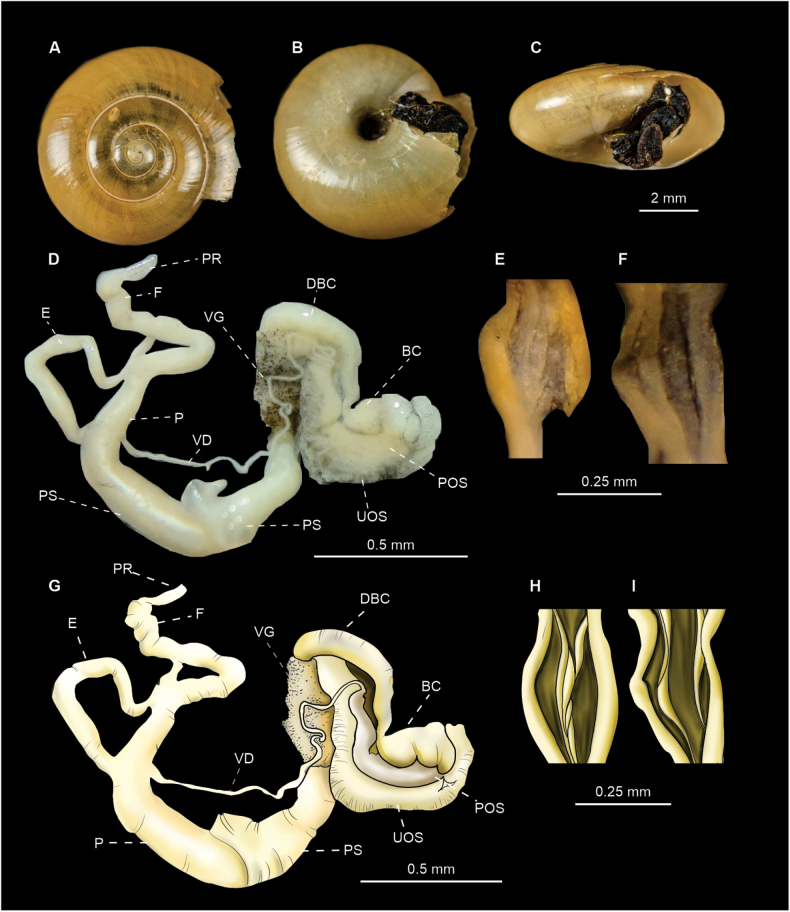
*Oxychilusalliarius* (CNMO 8464 Teopancingo, Puebla). **A–C** shell in apical, ventral and apertural view **D**, **G** genitalia of *O.alliarius***E, H** internal ornamentation of the proximal penis **F, I** internal ornamentation of the distal penis. Abbreviations: BC bursa copulatrix, DBC bursa duct, E epiphalus, F flagellum, P penis, POS prostatic portion of the ovospermiduct, PR penial retractor muscle, PS penial sheath, UOS uterine portion of the ovospermiduct, VG vaginal gland, VD vas deferens.

Two specimens were dissected. The proximal part of the penis is initially wider and then gradually becomes distally narrower as described by Giusti and Manganelli (2002) (Fig. 5D, G). Internally the proximal (Fig. 5E, H) and distal penis show 2–4, thin, slightly undulating, longitudinal folds that seem to be a continuation of one another, but never showing lateral branching or assuming a papilla-like shape (Fig. 5F, I).

***Shell*** discoidal, depressed, slightly convex above, compressed below, thin, semitransparent, variably shiny, yellowish to yellowish-brown, opalescent below (Fig. 5B, C); surface fairly smooth, with fainter growth lines more pronounced at sutures and very fine, wavy, spiral lines (Fig. 5A); aperture oval, oblique. Shell whorls: 4–4½. Umbilicus is rather broad, ~ 1⁄6 of maximum shell diameter. Shell dimensions: 6–9 mm diameter, 3–4 mm height (Fig. 7). Aperture width: 2–4 mm.

***Radula*** (*n* = 3) composed of~ 35 rows with 25–31 teeth/row (Fig. 8B). Radular formula: C/3 + 2–3 L/3 + 0–1 LM/2 + 9–13 M/1.

#### ﻿ Oxychilus cellarius

Taxon classificationAnimaliaStylommatophoraOxychilidae

(O.F. Müller, 1774)

96273467-2318-5FD5-AB5B-7DC8114DD428

Fig. 6

##### Worldwide distribution.

Originally described from a wine cellar in Copenhagen (Müller 1774). Its native range includes northern and western Europe (Horáčková and Juřičková 2009), Asia Minor, and North Africa (Roth and Sadeghian 2003). It has been introduced into Tasmania (Kershaw 1991), Greenland, North America (Forsyth 2004), St Helena, South Africa, Chile (Stuardo and Vega 1985), Hawaii (Cowie 1997), Australia and New Zealand (Barker 1999).

##### Distribution in Mexico.

CDMX (Tlalpan).

##### Diagnostic features.

**Figure 6.**
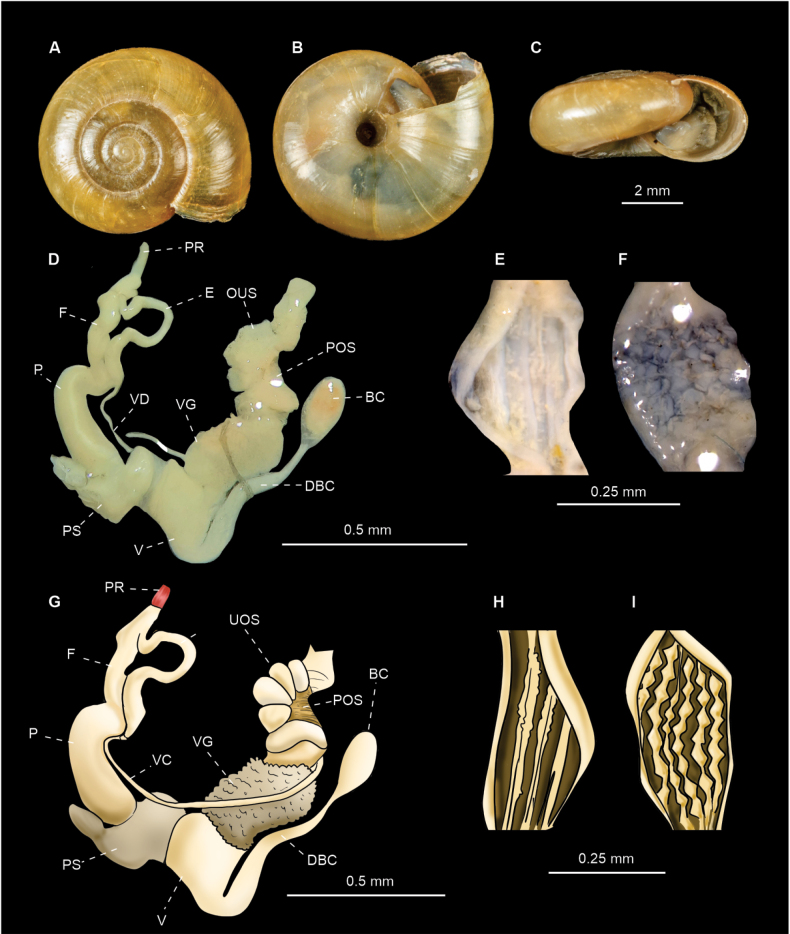
*Oxychiluscellarius* (CNMO 3858 Tlalpan, CDMX). **A–C** shell in apical, ventral and apertural view **D, G** genitalia of *O.cellarius***E, H** internal ornamentation of the distal penis **F, I** internal ornamentation of proximal penis. Abbreviations: BC bursa copulatrix, DBC bursa duct, E epiphalus, F flagellum, P penis, POS prostatic portion of the ovospermiduct, PR penial retractor muscle, PS penial sheath, UOS uterine portion of the ovospermiduct, V vagina, VD vas deferens, VG vaginal glands.

Two specimens were dissected. The penis is cylindrical with a relatively constant width in the middle portion (Fig. 6D, G). Internally the proximal penis shows small, evenly distributed papillae that tend to be joined together in rows which occasionally form undulating folds (Fig. 6F, I). The distal penis presents four, thin, internal folds (Fig. 6E, H).

**Figure 7.**
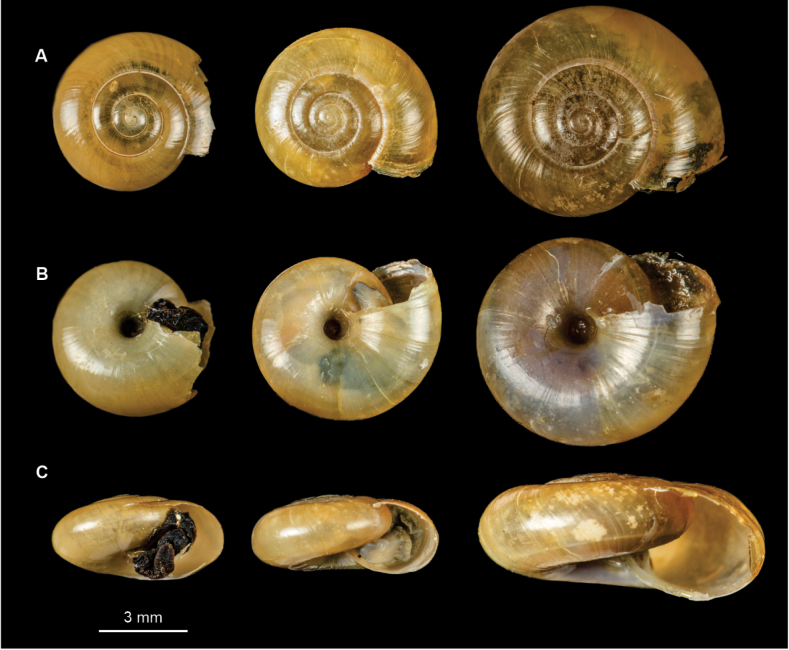
Shells of Mexican (from left to right) *Oxychilusalliarius* (CNMO 8464 Teopancingo, Puebla, *O.cellarius* (CNMO 3858 Tlalpan, CDMX), and *O.draparnaudi* (CNMO 8470 Cadereyta, Querétaro). **A** apical view **B** ventral view **C** apertural view.

***Shell*** discoidal, flattened, convex (Fig. 6C), light yellowish in color with fine lines of radial growth (Fig. 6A). Shell whorls: 4½–5. Umbilicus slightly flared, ~ 1/12 of maximum shell diameter (Fig. 6B). Shell dimensions: 8–10 mm diameter, 4 mm height (Fig. 7). Aperture width 3–4 mm.

**Figure 8.**
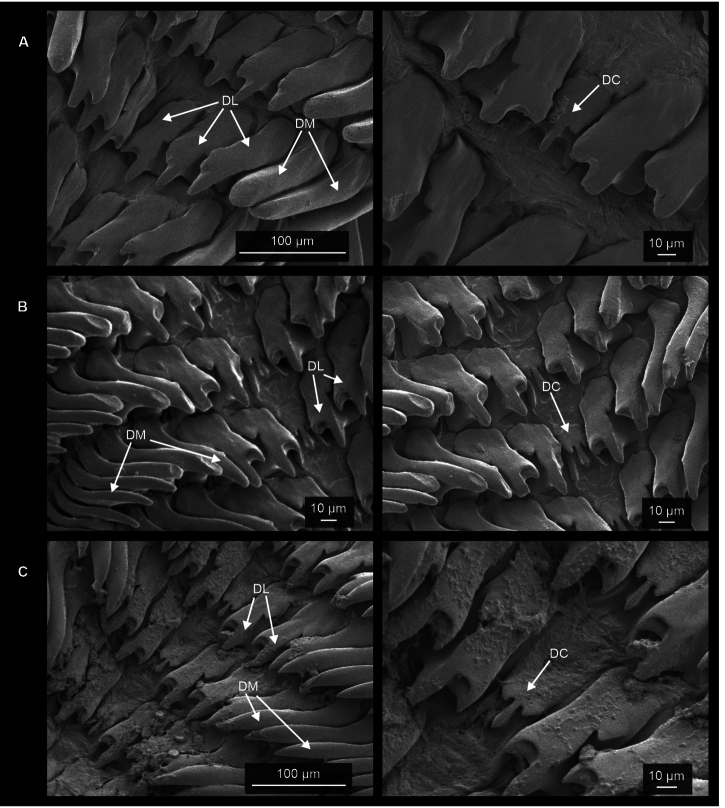
Radulae of Mexican. **A***O.draparnaudi* (CNMO 8470 Cadereyta, Querétaro), **B***O.alliarius* (CNMO 8464 Teopancingo, Puebla), and **C.***O.cellarius* (CNMO 3858 Tlalpan, CDMX). Abbreviations: DC central tooth, DL lateral teeth, DM marginal teeth.

***Radula*** (*n* = 2) composed of 35 rows with ~ 25 teeth/row (Fig. 8C). Radular formula: C/3+ 2–3 L/3+ 0–1 LM/2 + 9–14 M/1.

### ﻿mtDNA analysis

**Figure 9.**
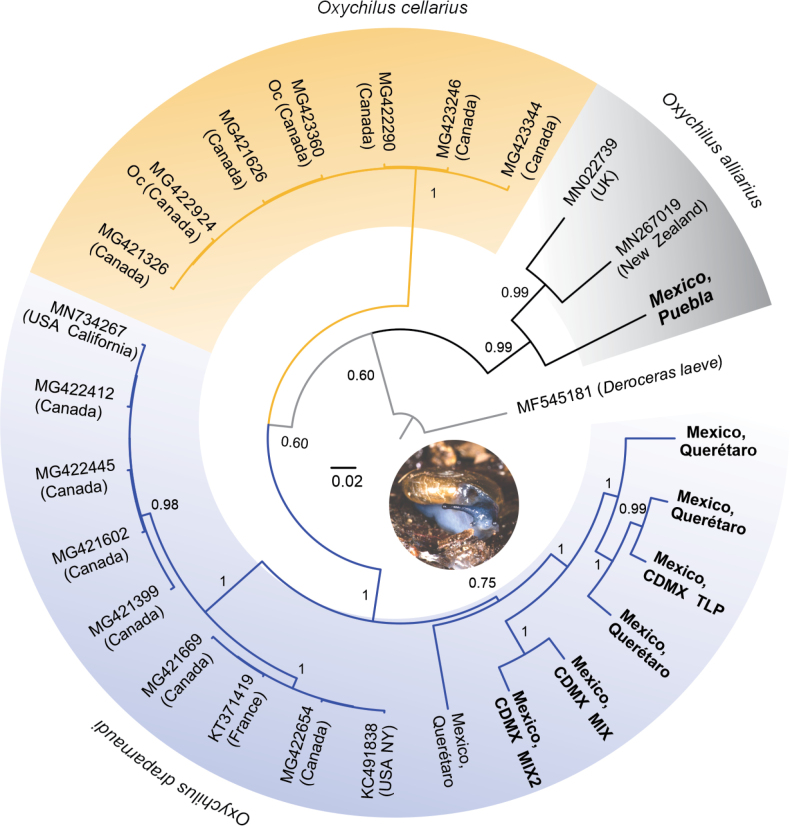
Bayesian phylogenetic tree of Mexican *Oxychilusalliarius*, *O.cellarius*, and *O.draparnaudi* based on the COI gene fragment (450 bp).

The COI tree recovered three clades corresponding to the three species studied in this study (Fig. 9), each with a posterior probability of 0.99–1.00. The sequence of the specimen from Teopancingo Puebla (TEN) was associated with GenBank sequences of *O.alliarius* from the United Kingdom and New Zealand, whose morphology agrees with that of the Mexican specimens. The other eight sequences from Querétaro, Tlaxcala, CDMX and Edo. Mex. (Suppl. material 1: table S3) formed a group with the GenBank sequences of *O.draparnaudi* from France, Canada, and the USA. Attempts to amplify the COI gene fragment for *O.cellarius* were unsuccessful due to insufficient DNA quality. However, the sequences reported in GenBank for this species were included in the analysis and formed a distinct group.

### ﻿Geometric morphometric analyses

**Figure 10.**
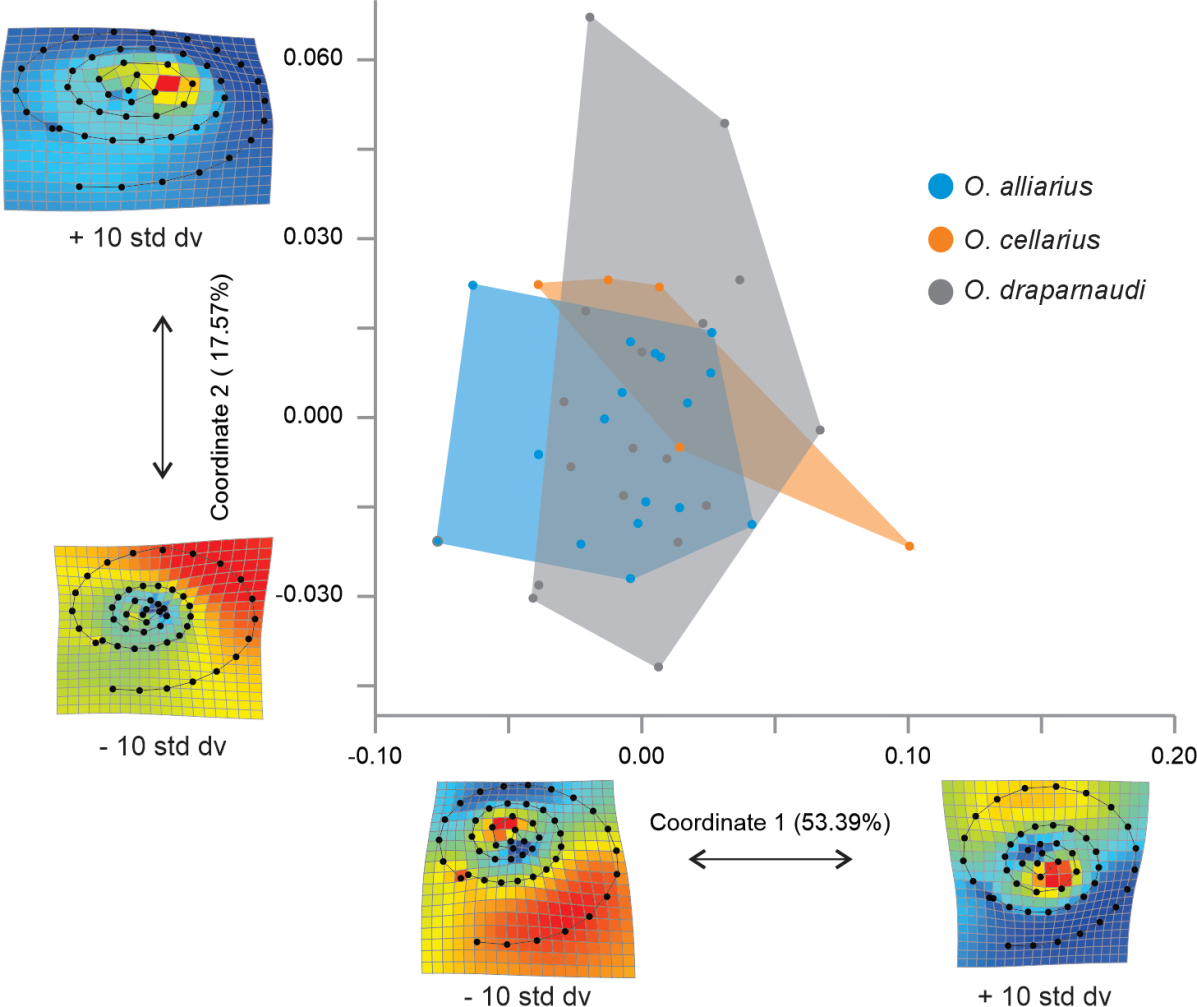
Geometric morphometric variation among shells of Mexican *Oxychilusalliarius*, *O.cellarius*, and *O.draparnaudi* in apical view.

**Figure 11.**
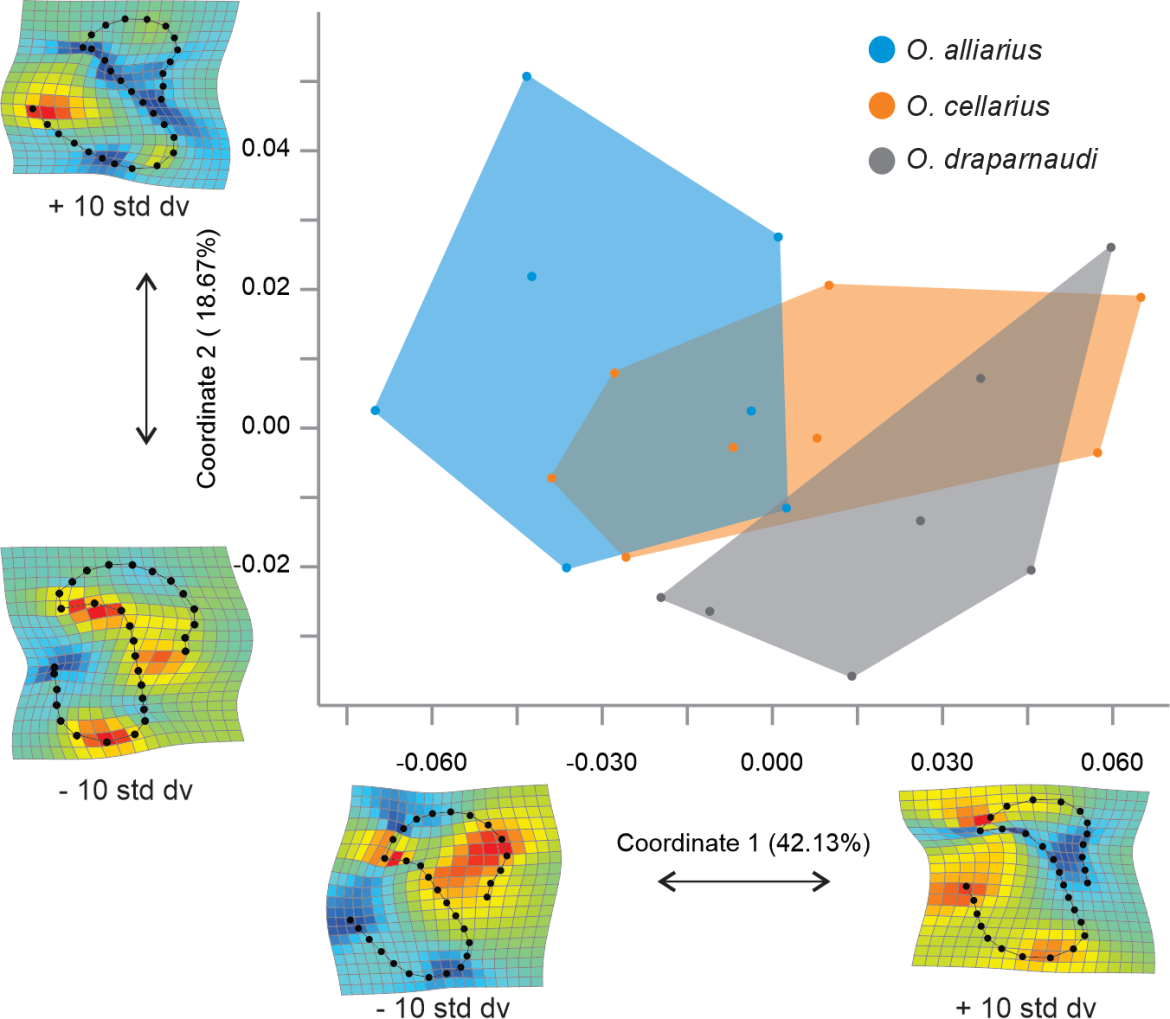
Geometric morphometric variation among shells of Mexican *Oxychilusalliarius*, *O.cellarius*, and *O.draparnaudi* in apertural view.

The superimposition of shell shape configuration in the apertural view for each individual did not exhibit regions with differences among the species. However, in the apical view, slight variations in the width of the shell aperture were observed (Fig. 9). The first two principal coordinates of the PCoA in the apertural view accounted for 70.96% of the variation (PCo1 = 53.39%, PCo2 = 17.57%), while in the apical view, they accounted for 60.80% of the variation (PCo1 = 42.13%, PCo2 = 18.67%). The scatterplots of the first two PCoA coordinates did not separate the species in either of the two evaluated views (Figs 10, 11).

### ﻿Distribution analysis

**Figure 12.**
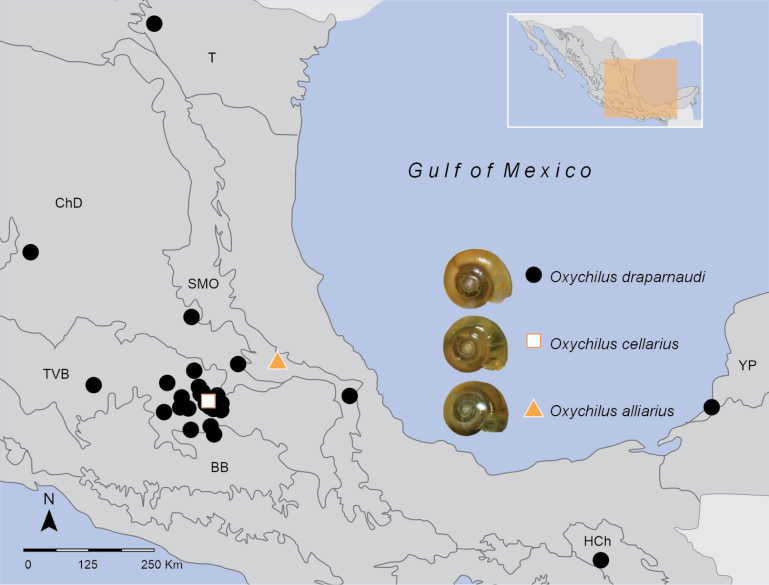
Actual distributions of *O.draparnaudi* (circle), *O.cellarius* (square), and *O.alliarius* (triangle) in Mexico. Biogeographic provinces (Morrone et al. 2017): T. Tamaulipas Province, ChD. Chihuahuan Desert Province SMO. Sierra Madre Oriental Province, TVB. Transmexican Volcanic Belt Province, BB. Balsas Basin Province, HCh. Chiapas Highlands Province, YP. Yucatan Peninsula Province.

**Figure 13.**
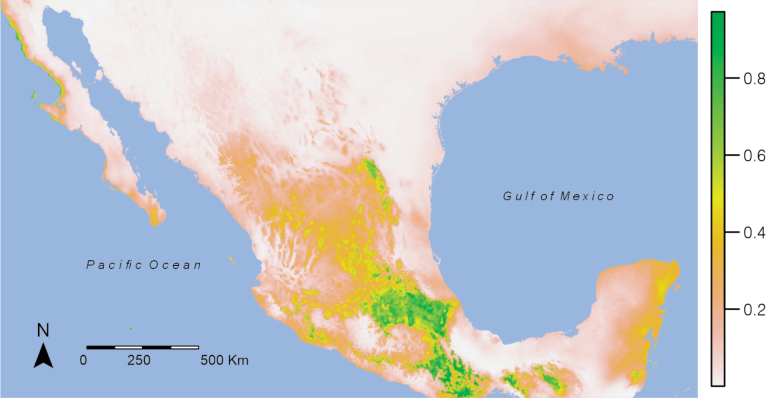
Potential distribution of the genus *Oxychilus* in Mexico. Green areas indicate high suitability areas.

A map was obtained using data from Mexican specimens of *O.alliarius*, *O.cellarius*, and *O.draparnaudi*, showing that *O.draparnaudi* is the most widespread of the three species. Most records are in the Transmexican Volcanic Belt Province, including the states of Querétaro, Puebla, CDMX, and the State of Mexico (Fig. 12). The potential distribution map for the genus *Oxychilus* shows a tendency towards locations in the Volcanic Axis, a part of the Eastern Sierra Madre, and a part of the Southern Sierra Madre (Fig. 13). The variables that contributed most for predicting potential areas according to correlation and jackknife analysis (Suppl. material 1: fig. S1) were BIO_5 (maximum temperature of warmest month), BIO_14 (precipitation of driest month (mm), BIO_11 (mean temperature of coldest quarter), BIO15 (precipitation seasonality (coefficient of variation)), BIO_3 (isothermality), BIO_7 (temperature annual range) and BIO_2 (mean diurnal range). The max temperature of the warmest month (BIO_5) of potential areas was 20–25 °C, and the precipitation during the driest month (Bio_ 14) was 5–15 mm (Suppl. material 1: fig. S2). The model performed well with an AUC of 0.958 (Suppl. material 1: fig. S3) and a TSS of 0.86.

## ﻿Discussion

The anatomical and DNA analyses unequivocally confirm the identification of *O.draparnaudi* and *O.alliarius*, and provide the first documentation of the invasive species *O.alliarius* in Mexico, while expanding the Mexican distribution range of *O.draparnaudi*. However, the potential distribution model indicates that the genus *Oxychilus* can be established not only in urban areas, but also in mountain regions, where *Oxychilus* species may potentially threaten native biodiversity. This was already observed with *O.alliarius* in natural areas like Hawaiian islands (Curry et al. 2016). Its presence is limited to temperate and humid regions, where temperatures are neither excessively high nor low, and where there is moderate water availability throughout the year. Sensitivity to desiccation and extreme temperatures are limiting factors to all the land snails (Nicolai and Ansart 2017). As such, isothermality (BIO_3) is an important climate variable that is commonly shared with other potential distribution models for land snails (Vogler et al. 2013; Adhikari et al. 2020; Horsák et al. 2022).

Although COI is an effective marker to identify *Oxychilus* species (Dahirel et al. 2015; Salvador et al. 2019), we could not apply it to Mexican *O.cellarius* because of the poor quality of the extracted DNA. However, the anatomical data were sufficiently evident to confirm the presence of this species in Mexico.

Given that in field conditions one may often find more empty shells than live animals, it is tempting to identify the three *Oxychilus* species by their shells only. However, our geometric morphometric analyses have shown that it is very tricky, if not impossible, to distinguish the three species conchologically. Hence, more accurate identification techniques are required. Usually, live *O.alliarius* are easily recognized by its characteristic garlic smell (Cádiz et al. 2013); however, we don´t detect that smell in the specimens reported in this study. Shell morphology did not show any identifiable pattern of variability, the three identified species show similarities, the only difference being size. *O.alliarius* is the species with the smallest size, ranging from 6 to 9 mm, followed by *O.cellarius* with a diameter between 8 and 10 mm, while *O.draparnaudi* is the largest, ranging from 9 mm to 13 mm in diameter. However, shell size is an unreliable diagnostic feature since it shows some overlap among the three species (Giusti and Manganelli 1997; Cádiz et al. 2013), particularly when immature specimens are involved. Hence, because of the conchological similarity between O. *alliarius*, *O.cellarius*, and *O.draparnaudi*, their identification should as much as possible be based on both their genital features and COI sequence data. Our results show that *O.draparnaudi* is already widespread in the central part of Mexico and may spread further southward. More research is required to determine to what extent local native faunas are impacted by *Oxychilus* species, since the three *Oxychilus* species reported here are partially carnivorous (Speiser 2001).

## Supplementary Material

XML Treatment for Oxychilus

XML Treatment for Oxychilus draparnaudi

XML Treatment for Oxychilus alliarius

XML Treatment for Oxychilus cellarius

## ﻿Additional information

### Author ORCIDs

Ali Gabrielle Trujillo-Díaz https://orcid.org/0009-0002-2120-6414

Victoria Araiza-Gómez https://orcid.org/0000-0001-5583-1918

Jazmín García-Román https://orcid.org/0000-0003-0044-339X

Gerardo Zúñiga https://orcid.org/0000-0002-5041-4258

Edna Naranjo-García https://orcid.org/0000-0001-9015-1758

### Data availability

All of the data that support the findings of this study are available in the main text or Supplementary Information.

## Supplementary materials

Supplementary material 1 Supplementary data Data type docx Explanation note Distribution data obtained in this study and consulted in databases used for potential distribution analysis are attached, in addition to the accession numbers of the COI gene sequences generated in this study. https://binary.pensoft.net/file/1236240This dataset is made available under the Open Database License (http://opendatacommons.org/licenses/odbl/1.0/). The Open Database License (ODbL) is a license agreement intended to allow users to freely share, modify, and use this Dataset while maintaining this same freedom for others, provided that the original source and author(s) are credited.Ali Gabrielle Trujillo-Díaz, Victoria Araiza-Gómez, Jazmín García-Román, José Luis Hernández-Domínguez, Gerardo Zúñiga, Edna Naranjo-García
