# Supplementary material for: ﻿First records of Oxychilusalliarius and O.cellarius (Gastropoda, Stylommatophora, Oxychilidae) in Mexico: mtDNA identification and potential distributions
Source: Zookeys. 2025 Jan 28;1224:141–64. doi: 10.3897/zookeys.1224.129618 (PMC11795182; doi:10.3897/zookeys.1224.129618)
Supplement: Supplementary material 1 — Supplementary data [file zookeys-1224-141_article-129618__-s001.docx]

**Supplementary files**

**Table 1.** Individuals collected and analyzed in this study.

| ID collection | ID | # organisms | Locality | Geographical coordinates | Species |
| --- | --- | --- | --- | --- | --- |
| CNMO8464 | TEN | 3 | Mexico, Puebla, Teopancingo | -98.07888°W, 20.09861°N | *O. alliarius* |
| CNMO8465 | TLA | 4 | Mexico, Edo. de Mexico, Tlalnepantla | -99.20694°W, 19.51500°N | *O. draparnaudi* |
| CNMO8466 | BT1 | 9 | Mexico, CDMX, Bosque de Tlalpan | -99.20333°W, 19.29111°N | *O. draparnaudi* |
| CNMO434 | OP | 8 | Mexico, CDMX, San Angel Olivar de los Padres | -99.21931°W, 19.33919°N | *O. draparnaudi* |
| CNMO3730 | BT2 | 2 | Mexico, CDMX, Bosque de Tlalpan | -99.19856°W, 19.29379°N | *O. draparnaudi* |
| CNMO3857 | BT3 | 2 | Mexico, CDMX, Bosque de Tlalpan | -99.19856°W, 19.29379°N | *O. draparnaudi* |
| CNMO3858 | BT4 | 2 | Mexico, CDMX, Bosque de Tlalpan | -99.19856°W, 19.29379°N | *O. cellarius* |
| CNMO8467 | MIX1 | 2 | Mexico, CDMX, Mixcoac | -99.189855°W, 19.37281°N | *O. draparnaudi* |
| CNMO8468 | MIX2 | 3 | Mexico, CDMX, Mixcoac | -99.189855°W, 19.37281°N | *O. draparnaudi* |
| CNMO8469 | MIX3 | 2 | Mexico, CDMX, Mixcoac | -99.189855°W, 19.37281°N | *O. draparnaudi* |
| CNMO8470 | EC1 | 1 | Mexico, Queretaro, Cadereyta de Montes, Maconí. El Caracol | -99.539186°W, 20.844502°N | *O. draparnaudi* |
| CNMO8470 | EC2 | 1 | Mexico, Queretaro, Cadereyta de Montes, Maconí. El Caracol | -99.539186°W, 20.844502°N | *O. draparnaudi* |
| CNMO8470 | EC3 | 1 | Mexico, Queretaro, Cadereyta de Montes, Maconí. El Caracol | -99.539186°W, 20.844502°N | *O. draparnaudi* |
| CNMO8470 | EC4 | 1 | Mexico, Queretaro, Cadereyta de Montes, Maconí. El Caracol | -99.539186°W, 20.844502°N | *O. draparnaudi* |
| CNMO8470 | EC5 | 1 | Mexico, Queretaro, Cadereyta de Montes, Maconí. El Caracol | -99.539186°W, 20.844502°N | *O. draparnaudi* |
| CNMO8471 | ATL | 1 | Mexico, Tlaxcala, Atlihuetzia | -98.177222°W, 19.37222°N | *O. draparnaudi* |
| CNMO8473 | ATL2 | 3 | Mexico, Tlaxcala, Atlihuetzia | -98.177222°W, 19.37222°N | *O. draparnaudi* |
| CNMO8472 | ATL3 | 4 | Mexico, Tlaxcala, Atlihuetzia | -98.177222°W, 19.37222°N | *O. draparnaudi* |

**Table 2.** Distribution data.

| *Genus* | Species | Longitud | Latitud | Source | Locality |
| --- | --- | --- | --- | --- | --- |
| *Oxychilus* | *Oxychilus* sp. | -98.080278 | 20.09861 | CNMO | México, Puebla |
| *Oxychilus* | *Oxychilus* sp. | -99.20704 | 19.51575 | CNMO | Edo. Mex., Tlalnepantla |
| *Oxychilus* | *Oxychilus draparnaudi* | -99.20333 | 19.29111 | CNMO | CDMX, Tlalpan |
| *Oxychilus* | *Oxychilus draparnaudi* | -99.219316 | 19.339197 | CNMO | CDMX, Olivar de los Padres |
| *Oxychilus* | *Oxychilus draparnaudi* | -99.19856 | 19.293797 | CNMO | CDMX, Tlalpan |
| *Oxychilus* | *Oxychilus* sp. | -99.19856 | 19.293797 | CNMO | CDMX, Tlalpan |
| *Oxychilus* | *Oxychilus draparnaudi* | -99.189855 | 19.372818 | CNMO | CDMX, Mixcoac |
| *Oxychilus* | *Oxychilus draparnaudi* | -99.189855 | 19.372818 | CNMO | CDMX, Mixcoac |
| *Oxychilus* | *Oxychilus* sp. | -99.189855 | 19.372818 | CNMO | CDMX, Mixcoac |
| *Oxychilus* | *Oxychilus draparnaudi* | -99.539186 | 20.844502 | CNMO | Querétaro |
| *Oxychilus* | *Oxychilus draparnaudi* | -99.539186 | 20.844502 | CNMO | Querétaro |
| *Oxychilus* | *Oxychilus draparnaudi* | -99.539186 | 20.844502 | CNMO | Querétaro |
| *Oxychilus* | *Oxychilus draparnaudi* | -98.177391 | 19.373205 | CNMO | Tlaxcala, Atlihuetzia |
| *Oxychilus* | *Oxychilus draparnaudi* | -99.539186 | 20.844502 | CNMO | Querétaro |
| *Oxychilus* | *Oxychilus draparnaudi* | -99.192182 | 19.315331 | Rivera García A. 2013. Malacofauna terrestre del Pedregal de San Ángel, Núcleo Poniente. Tesis para obtener el título de biólogo. UNAM | CDMX, Coyoacán |
| *Oxychilus* | *Oxychilus draparnaudi* | -99.19657 | 19.3185 | Rivera García A. 2013. Malacofauna terrestre del Pedregal de San Ángel, Núcleo Poniente. Tesis para obtener el título de biólogo. UNAM | CDMX, Coyoacán |
| *Oxychilus* | *Oxychilus draparnaudi* | -99.192182 | 19.315331 | Rivera García A. 2013. Malacofauna terrestre del Pedregal de San Ángel, Núcleo Poniente. Tesis para obtener el título de biólogo. UNAM | CDMX, Coyoacán |
| *Oxychilus* | *Oxychilus draparnaudi* | -99.19155 | 19.31947 | Rivera García A. 2013. Malacofauna terrestre del Pedregal de San Ángel, Núcleo Poniente. Tesis para obtener el título de biólogo. UNAM | CDMX, Coyoacán |
| *Oxychilus* | *Oxychilus draparnaudi* | -99.192182 | 19.315331 | Rivera García A. 2013. Malacofauna terrestre del Pedregal de San Ángel, Núcleo Poniente. Tesis para obtener el título de biólogo. UNAM | CDMX, Coyoacán |
| *Oxychilus* | *Oxychilus draparnaudi* | -99.200554 | 19.310485 | Rivera García A. 2013. Malacofauna terrestre del Pedregal de San Ángel, Núcleo Poniente. Tesis para obtener el título de biólogo. UNAM | CDMX, Coyoacán |
| *Oxychilus* | *Oxychilus draparnaudi* | -99.1933 | 19.31338 | Rivera García A. 2013. Malacofauna terrestre del Pedregal de San Ángel, Núcleo Poniente. Tesis para obtener el título de biólogo. UNAM | CDMX, Coyoacán |
| *Oxychilus* | *Oxychilus draparnaudi* | -99.200554 | 19.310485 | Rivera García A. 2013. Malacofauna terrestre del Pedregal de San Ángel, Núcleo Poniente. Tesis para obtener el título de biólogo. UNAM | CDMX, Coyoacán |
| *Oxychilus* | *Oxychilus draparnaudi* | -99.194145 | 19.32073 | Rivera García A. 2013. Malacofauna terrestre del Pedregal de San Ángel, Núcleo Poniente. Tesis para obtener el título de biólogo. UNAM | CDMX, Coyoacán |
| *Oxychilus* | *Oxychilus draparnaudi* | -99.19372 | 19.313655 | Rivera García A. 2013. Malacofauna terrestre del Pedregal de San Ángel, Núcleo Poniente. Tesis para obtener el título de biólogo. UNAM | CDMX, Coyoacán |
| *Oxychilus* | *Oxychilus draparnaudi* | -99.195192 | 19.314321 | Rivera García A. 2013. Malacofauna terrestre del Pedregal de San Ángel, Núcleo Poniente. Tesis para obtener el título de biólogo. UNAM | CDMX, Coyoacán |
| *Oxychilus* | *Oxychilus draparnaudi* | -99.195192 | 19.314321 | Rivera García A. 2013. Malacofauna terrestre del Pedregal de San Ángel, Núcleo Poniente. Tesis para obtener el título de biólogo. UNAM | CDMX, Coyoacán |
| *Oxychilus* | *Oxychilus draparnaudi* | -99.19857 | 19.293797 | GBIF-inaturalist | CDMX, Tlalpan |
| *Oxychilus* | *Oxychilus draparnaudi* | -99.653711 | 19.293573 | GBIF-inaturalist | Mexico, Toluca |
| *Oxychilus* | *Oxychilus draparnaudi* | -100.50304 | 25.869432 | GBIF-inaturalist | Nuevo León, García |
| *Oxychilus* | *Oxychilus draparnaudi* | -99.95599 | 19.716084 | GBIF-inaturalist | México, San Felipe del progreso, Valle de Toluca |
| *Oxychilus* | *Oxychilus draparnaudi* | -99.245346 | 19.400928 | GBIF-inaturalist | CDMX, Miguel Hidalgo |
| *Oxychilus* | *Oxychilus draparnaudi* | -100.013565 | 19.251233 | GBIF-inaturalist | Amanalco |
| *Oxychilus* | *Oxychilus draparnaudi* | -99.195991 | 19.292143 | GBIF-inaturalist | CDMX, Tlalpan |
| *Oxychilus* | *Oxychilus draparnaudi* | -99.182291 | 19.284385 | GBIF-inaturalist | CDMX, Tlalpan, Fuentes brotantes |
| *Oxychilus* | *Oxychilus draparnaudi* | -101.200027 | 19.672788 | GBIF-inaturalist | Morelia |
| *Oxychilus* | *Oxychilus draparnaudi* | -99.138856 | 19.308503 | GBIF-inaturalist | Coyoacán, Ex-Hacienda Coapa |
| *Oxychilus* | *Oxychilus draparnaudi* | -117.018495 | 32.529791 | GBIF-inaturalist | Baja California, Tijuana. Frontera BC |
| *Oxychilus* | *Oxychilus draparnaudi* | -99.188566 | 19.422742 | GBIF-inaturalist | CDMX, Miguel Hidalgo |
| *Oxychilus* | *Oxychilus draparnaudi* | -99.280384 | 19.372206 | GBIF-inaturalist | CDMX, Cuajimalpa |
| *Oxychilus* | *Oxychilus draparnaudi* | -99.070015 | 19.549703 | GBIF-inaturalist | México, Ecatepec de Morelos |
| *Oxychilus* | *Oxychilus draparnaudi* | -99.168568 | 19.34163 | GBIF-inaturalist | CDMX, Coyoacán |
| *Oxychilus* | *Oxychilus draparnaudi* | -99.049652 | 19.263111 | GBIF-inaturalist | CDMX,Tlalpan |
| *Oxychilus* | *Oxychilus draparnaudi* | -99.200805 | 19.330808 | GBIF-inaturalist | CDMX, Álvaro Obregón |
| *Oxychilus* | *Oxychilus draparnaudi* | -99.235052 | 19.330311 | GBIF-inaturalist | CDMX, Álvaro Obregón |
| *Oxychilus* | *Oxychilus draparnaudi* | -98.737751 | 20.07413 | GBIF-inaturalist | Hidalgo, Mineral de Reforma |
| *Oxychilus* | *Oxychilus draparnaudi* | -98.73848 | 20.077019 | GBIF-inaturalist | Hidalgo, Mineral de Reforma |
| *Oxychilus* | *Oxychilus draparnaudi* | -98.73848 | 20.077019 | GBIF-inaturalist | Hidalgo, Mineral de Reforma |
| *Oxychilus* | *Oxychilus draparnaudi* | -99.7008 | 18.550669 | GBIF-inaturalist | Guerrero, Taxco de Alarcón |
| *Oxychilus* | *Oxychilus draparnaudi* | -98.73848 | 20.077019 | GBIF-inaturalist | Hidalgo, Mineral de Reforma |
| *Oxychilus* | *Oxychilus draparnaudi* | -99.52517 | 19.933788 | GBIF-inaturalist | México, Jilotepec |
| *Oxychilus* | *Oxychilus draparnaudi* | -99.095286 | 19.869491 | GBIF-inaturalist | México, Zumpango |
| *Oxychilus* | *Oxychilus draparnaudi* | -99.197908 | 19.29284 | GBIF-inaturalist | CDMX, Tlalpan |
| *Oxychilus* | *Oxychilus draparnaudi* | -99.735299 | 19.446817 | GBIF-inaturalist | Almoloya de Juárez, Fraccionamiento Rancho San Juan |
| *Oxychilus* | *Oxychilus draparnaudi* | -102.298706 | 21.939796 | GBIF-inaturalist | Aguascalientes, Residencial Cedros |
| *Oxychilus* | *Oxychilus draparnaudi* | -99.141739 | 19.259261 | GBIF-inaturalist | CDMX, Xochimilco |
| *Oxychilus* | *Oxychilus draparnaudi* | -92.631827 | 16.737911 | GBIF-inaturalist | Chiapas, San Cristóbal |
| *Oxychilus* | *Oxychilus draparnaudi* | -99.243492 | 18.980479 | GBIF-inaturalist | Morelos, Cuernavaca |
| *Oxychilus* | *Oxychilus draparnaudi* | -99.632751 | 19.314556 | GBIF-inaturalist | Toluca, San Lorenzo |
| *Oxychilus* | *Oxychilus draparnaudi* | -92.634355 | 16.704162 | GBIF-inaturalist | Chiapas, San Cristóbal |
| *Oxychilus* | *Oxychilus draparnaudi* | -92.634422 | 16.704223 | GBIF-inaturalist | Chiapas, San Cristóbal |
| *Oxychilus* | *Oxychilus draparnaudi* | -99.583878 | 18.962618 | GBIF-inaturalist | Puebla, Tenancingo |
| *Oxychilus* | *Oxychilus draparnaudi* | -90.731826 | 19.344067 | GBIF-inaturalist | Campeche, Champotón |
| *Oxychilus* | *Oxychilus draparnaudi* | -99.253597 | 19.318211 | GBIF-inaturalist | CDMX, Magdalena Contreras |
| *Oxychilus* | *Oxychilus draparnaudi* | -99.025183 | 19.407825 | GBIF-inaturalist | Edo. Mex., Nezahualcoyotl |
| *Oxychilus* | *Oxychilus draparnaudi* | -99.179855 | 19.325192 | GBIF-inaturalist | CDMX, Coyoacán |
| *Oxychilus* | *Oxychilus draparnaudi* | -99.193331 | 19.31987 | GBIF-inaturalist | CDMX, Coyoacán, Reserva el Pedregal |
| *Oxychilus* | *Oxychilus draparnaudi* | -99.194428 | 19.316862 | GBIF-inaturalist | CDMX, Coyoacán, Reserva el Pedregal |
| *Oxychilus* | *Oxychilus draparnaudi* | -99.257079 | 19.48384 | GBIF-inaturalist | Edo. Mex., Naucalpan |
| *Oxychilus* | *Oxychilus draparnaudi* | -99.175613 | 19.305925 | GBIF-inaturalist | CDMX, Coyoacán, Panamericana, Pedregal |
| *Oxychilus* | *Oxychilus draparnaudi* | -99.198682 | 19.291966 | GBIF-inaturalist | CDMX, Tlalpan, Camino a Sta. Teresa |
| *Oxychilus* | *Oxychilus draparnaudi* | -99.367551 | 19.633692 | GBIF-inaturalist | CDMX, Nicolás Romero |
| *Oxychilus* | *Oxychilus draparnaudi* | -99.187225 | 19.316389 | GBIF-inaturalist | CDMX, Coyoacán, Insurgentes Sur |
| *Oxychilus* | *Oxychilus draparnaudi* | -99.258857 | 19.483909 | GBIF-inaturalist | Edo. Mex., Naucalpan |
| *Oxychilus* | *Oxychilus draparnaudi* | -99.323044 | 19.577137 | GBIF-inaturalist | Edo. Mex., Atizapán de Zaragoza |
| *Oxychilus* | *Oxychilus draparnaudi* | -99.197136 | 19.296253 | GBIF-inaturalist | CDMX, Bosque de Tlalpan |
| *Oxychilus* | *Oxychilus draparnaudi* | -99.193029 | 19.328714 | GBIF-inaturalist | CDMX, Coyoacán |
| *Oxychilus* | *Oxychilus draparnaudi* | -99.017822 | 19.2586 | GBIF-inaturalist | CDMX, Xochimilco |
| *Oxychilus* | *Oxychilus draparnaudi* | -99.176132 | 19.323203 | GBIF-inaturalist | CDMX, Coyoacán |
| *Oxychilus* | *Oxychilus draparnaudi* | -99.199781 | 18.873338 | GBIF-inaturalist | Morelos, Emiliano Zapata |
| *Oxychilus* | *Oxychilus draparnaudi* | -99.19951 | 19.292116 | GBIF-inaturalist | CDMX, Tlalpan |
| *Oxychilus* | *Oxychilus* sp. | -86.81348 | 21.18254 | iNaturalistMx | Cancún QRO, Laguna de Manatí |
| *Oxychilus* | *Oxychilu*s sp. | -99.1179 | 19.56253 | iNaturalistMx | CDMX, Sierra de Guadalupe |
| *Oxychilus* | *Oxychilus* sp. | -100.75972 | 20.14437 | iNaturalistMx | Guanajuato, Acámbaro |
| *Oxychilus* | *Oxychilus* sp. | -99.14275 | 19.25774 | iNaturalistMx | CDMX, Tlalpan |
| *Oxychilus* | Oxychilus sp. | -92.63762 | 16.73704 | iNaturalistMx | Chiapas, San Cristóbal de las Casas |
| *Oxychilus* | Oxychilus sp. | -99.41071 | 19.56178 | iNaturalistMx | Edo. Mex., Tlanepantla de Baz, Bosque de agua, Isidro Fabela |
| *Oxychilus* | Oxychilus sp. | -99.58644 | 19.3585 | iNaturalistMx | México, Toluca |
| *Oxychilus* | Oxychilus sp. | -99.19394 | 19.28983 | iNaturalistMx | CDMX, Tlalpan |
| *Oxychilus* | Oxychilus sp. | -99.202 | 19.28901 | iNaturalistMx | CDMX, Tlalpan |
| *Oxychilus* | Oxychilus sp. | -99.15633 | 19.32596 | iNaturalistMx | CDMX, Coyoacán |
| *Oxychilus* | Oxychilus sp. | -100.07838 | 19.15304 | iNaturalistMx | México, Valle de Bravo |
| *Oxychilus* | Oxychilus sp. | -99.19636 | 19.29289 | iNaturalistMx | CDMX, Tlalpan |
| *Oxychilus* | Oxychilus sp. | -99.19656 | 19.29281 | iNaturalistMx | CDMX, Tlapan |
| *Oxychilus* | Oxychilus sp. | -99.19673 | 19.29272 | iNaturalistMx | CDMX, Parque bosque del pedregal, Tlalpan |
| *Oxychilus* | Oxychilus sp. | -117.02674 | 32.47554 | iNaturalistMx | Baja California, Tijuana |
| *Oxychilus* | Oxychilus sp. | -105.76651 | 24.08007 | iNaturalistMx | Durango, San Dimas |
| *Oxychilus* | Oxychilus sp. | -99.21185 | 19.36802 | iNaturalistMx | CDMX, Álvaro Obregón |
| *Oxychilus* | Oxychilus sp. | -99.1964 | 19.25155 | iNaturalistMx | CDMX, Tlalpan |
| *Oxychilus* | Oxychilus sp. | -99.23613 | 19.32886 | iNaturalistMx | CDMX, Álvaro Obregón, La loma |
| *Oxychilus* | Oxychilus sp. | -99.11815 | 19.5625 | iNaturalistMx | CDMX, GAM |
| *Oxychilus* | Oxychilus sp. | -99.12011 | 19.55789 | iNaturalistMx | CDMX, GAM |
| *Oxychilus* | Oxychilus sp. | -99.06669 | 19.33601 | iNaturalistMx | CDMX, Iztapalapa |
| *Oxychilus* | Oxychilus sp. | -92.66209 | 16.72296 | iNaturalistMx | Chiapas, San Cristóbal de las Casas |
| *Oxychilus* | Oxychilus sp. | -116.6665 | 31.86895 | iNaturalistMx | Baja California, Ensenada |
| *Oxychilus* | *Oxychilus* sp. | -99.19548 | 19.29024 | iNaturalistMx | CDMX, Tlalpan |
| *Oxychilus* | *Oxychilus sp.* | -99.31261 | 19.00833 | iNaturalistMx | Morelos, Cuernavaca |
| *Oxychilus* | *Oxychilus* sp. | -99.14203 | 19.25787 | iNaturalistMx | CDMX, Tlalpan |
| *Oxychilus* | *Oxychilus* sp. | -99.14214 | 19.25782 | iNaturalistMx | CDMX, Tlalpan |
| *Oxychilus* | *Oxychilus sp.* | -99.19495 | 19.2538 | iNaturalistMx | CDMX, Tlalpan |
| *Oxychilus* | *Oxychilus* sp. | -99.68404 | 19.87858 | iNaturalistMx | México, Timilpan, Presa Huapango |
| *Oxychilus* | *Oxychilus* sp. | -97.44422 | 20.95732 | iNaturalistMx | Veracruz, Tuxpan |
| *Oxychilus* | O*xychilu*s sp. | -99.19857 | **19.2938** | iNaturalistMx | CDMX, Tlalpan |
| *Oxychilus* | *Oxychilus* sp. | -99.13618 | 19.25952 | iNaturalistMx | CDMX, Xochimilco |
| *Oxychilus* | *Oxychilus sp.* | -99.19633 | 19.29357 | iNaturalistMx | CDMX, Tlalpan |
| *Oxychilus* | *Oxychilus sp.* | -100.01481 | 19.25689 | iNaturalistMx | Metepec, Amanalco |
| *Oxychilus* | *Oxychilus* sp. | -100.01345 | 19.2518 | iNaturalistMx | Amanalco |
| *Oxychilus* | *Oxychilus* sp. | -100.01559 | 19.25254 | iNaturalistMx | Amanalco |
| *Oxychilus* | *Oxychilu*s sp. | -92.62932 | 16.70702 | iNaturalistMx | Chiapas, San Cristobal de las Casas |
| *Oxychilus* | *Oxychilus* sp. | -99.2708 | 19.54953 | iNaturalistMx | Edo. Mex., Atizapán de Zaragoza |
| *Oxychilus* | *Oxychilus sp.* | -99.20157 | 19.29204 | iNaturalistMx | CDMX, Tlalpan |
| *Oxychilus* | *Oxychilus* sp. | -99.20151 | 19.29206 | iNaturalistMx | CDMX, Tlalpan |
| *Oxychilus* | *Oxychilus* sp. | -99.1914 | 19.33793 | iNaturalistMx | CDMX, Alvaro Obregón, Panteón San Rafael |
| *Oxychilus* | *Oxychilu*s sp. | -99.19619 | 19.29449 | iNaturalistMx | CDMX, Tlalpan |
| *Oxychilus* | *Oxychilu*s sp. | -101.20003 | 19.67279 | iNaturalistMx | Michoacán, Morelia |
| *Oxychilus* | *Oxychilu*s sp. | -99.23383 | 19.3684 | iNaturalistMx | CDMX, Álvaro Obregón |
| *Oxychilus* | Oxychilus sp. | -99.19786 | 19.29279 | iNaturalistMx | CDMX, Tlalpan |
| *Oxychilus* | Oxychilus sp. | -101.20036 | 19.67325 | iNaturalistMx | Michoacán, Morelia |
| *Oxychilus* | Oxychilus sp. | -99.20334 | 19.27057 | iNaturalistMx | CDMX, Tlalpan |
| *Oxychilus* | Oxychilus sp. | -99.70225 | 19.2983 | iNaturalistMx | México, Valle de Toluca |
| *Oxychilus* | Oxychilus sp. | -99.95145 | 19.71261 | iNaturalistMx | México, San Felipe del Progreso |
| *Oxychilus* | Oxychilus sp. | -99.33253 | 19.54602 | iNaturalistMx | México, Atizapán de Zaragoza |
| *Oxychilus* | Oxychilus sp. | -98.76239 | 20.0911 | iNaturalistMx | Hidalgo, Pachuca |
| *Oxychilus* | Oxychilus sp. | -96.86184 | 19.51327 | iNaturalistMx | Veracruz, Xalapa |
| *Oxychilus* | Oxychilus sp. | -99.14074 | 19.25687 | iNaturalistMx | CDMX, Xochimilco |
| *Oxychilus* | Oxychilus sp. | -99.14235 | 19.2603 | iNaturalistMx | CDMX, Xochimilco |
| *Oxychilus* | Oxychilus sp. | -99.13528 | 19.25983 | iNaturalistMx | CDMX, Xochimilco |
| *Oxychilus* | Oxychilus sp. | -99.17592 | 19.43536 | iNaturalistMx | CDMX, Miguel Hidalgo |
| *Oxychilus* | Oxychilus sp. | -99.55747 | 19.17334 | iNaturalistMx | México, Valle de Toluca |
| *Oxychilus* | Oxychilus sp. | -99.19598 | 19.29196 | iNaturalistMx | CDMX, Tlalpan |
| *Oxychilus* | Oxychilus sp. | -99.25739 | 19.48613 | iNaturalistMx | Edo. Mex., Naucalpan |
| *Oxychilus* | Oxychilus sp. | -99.81275 | 20.69528 | iNaturalistMx | Querétaro, Cadereyta |
| *Oxychilus* | Oxychilus draparnaudi | -99.17898 | 19.3243 | GBIF (Carnegie Museum of Natural History) | CDMX, UNAM Tlahuizcalpan building |

**Table 3.** Accession number of the sequences generated in this study.

| **ID** | **Specie** | **Specimen Catalog #** | **GenBank # COI** |
| --- | --- | --- | --- |
| TEN | *Oxychilus alliarius* | CNMO 8464 | PP942456 |
| ATL | *Oxychilus draparnaudi* | CNMO 8471 | PP658224 |
| EC2 | *Oxychilus draparnaudi* | CNMO 8470 | PP431571 |
| EC3 | *Oxychilus draparnaudi* | CNMO 8470 | PP431573 |
| EC4 | *Oxychilus draparnaudi* | CNMO 8470 | PP431572 |
| EC5 | *Oxychilus draparnaudi* | CNMO 8470 | PP431574 |
| MIX1 | *Oxychilus draparnaudi* | CNMO 8467 | PP658222 |
| MIX2 | *Oxychilus draparnaudi* | CNMO 8468 | PP658223 |
| TLA (129) | *Oxychilus draparnaudi* | CNMO 8465 | PP431570 |


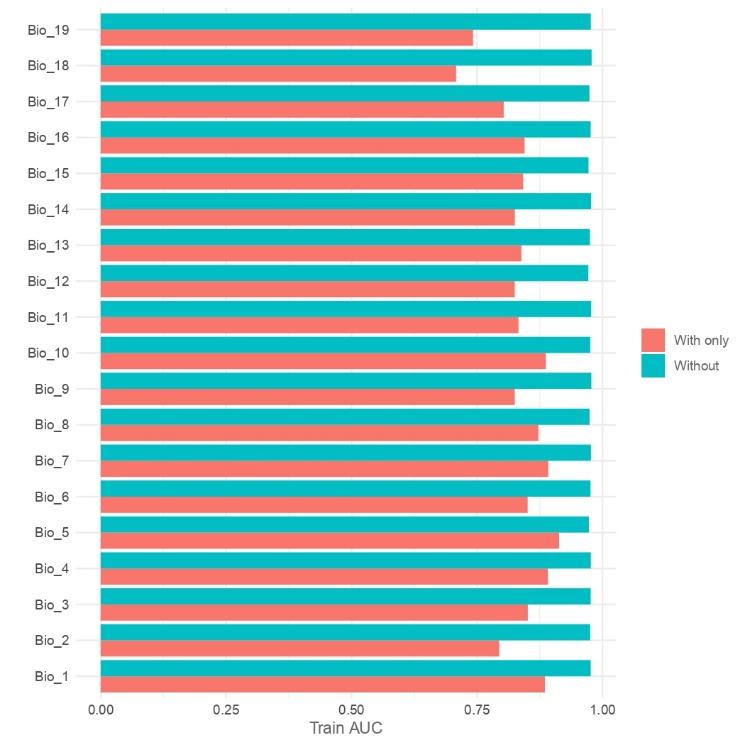


Figure S1. Jackknife test for variables used in the initial Maxent model to estimate potential distribution.


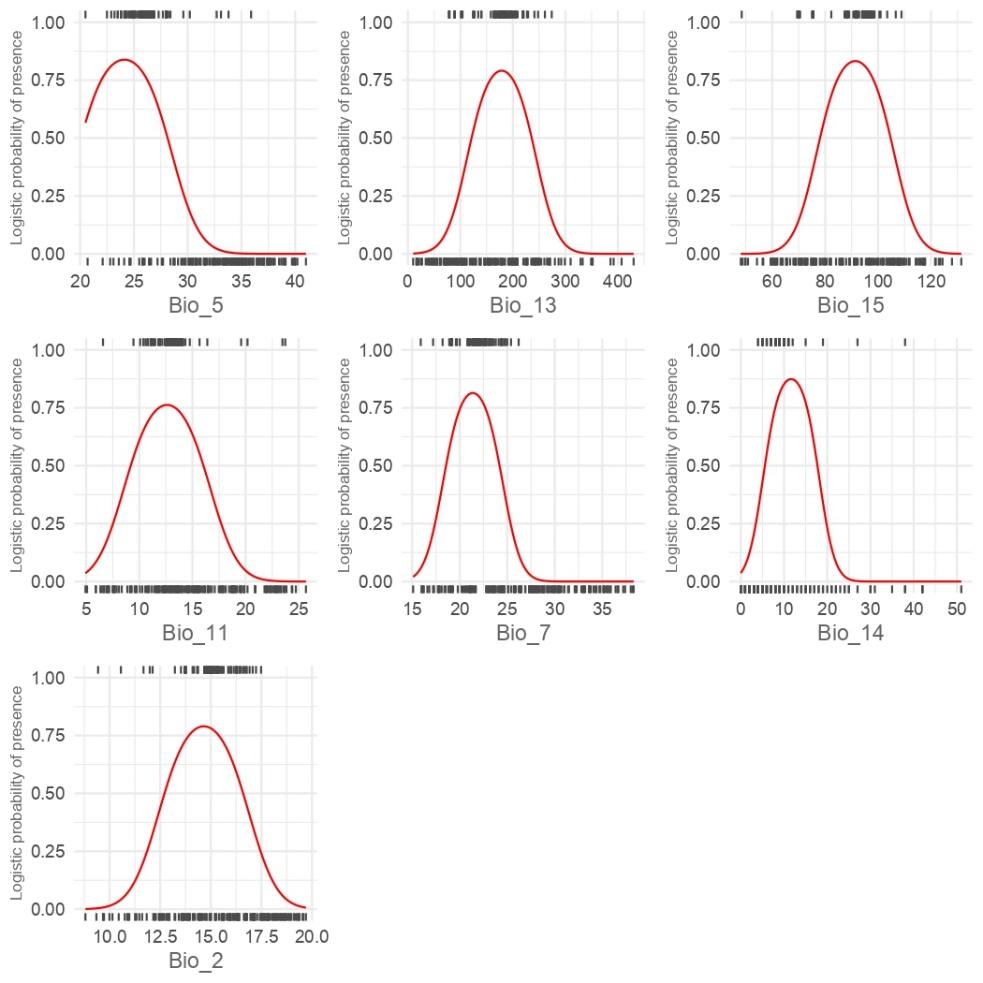


Figure S2. Response curves of variables potential distribution model, Nepal: Bio_05=Max Temperature of Warmest Month indicates the highest temperature of any monthly maximum temperature; Bio_14=precipitation of driest month (mm) refers to the precipitation of the driest month; Bio_11= Mean Temperature of Coldest Quarter indicates he coldest quarter of the year is determined (to the nearest month), and the mean temperature of this period is calculated; Bio_15= Precipitation Seasonality (Coefficient of Variation) is the coefficient of variation the standard deviation of the monthly precipitation estimates expressed as a percentage of the mean of those estimates; Bio_3 isothermality (°C) refers the mean diurnal range dividen by the annual temperature range; Bio_7= Temperature Annual Range (BIO5-BIO6) is the difference between the maximum temperature of warmest period and the minimum temperature of the coldest period; Bio_2=Mean Diurnal Range refers to the annual mean of all the monthly diurnal temperature ranges.


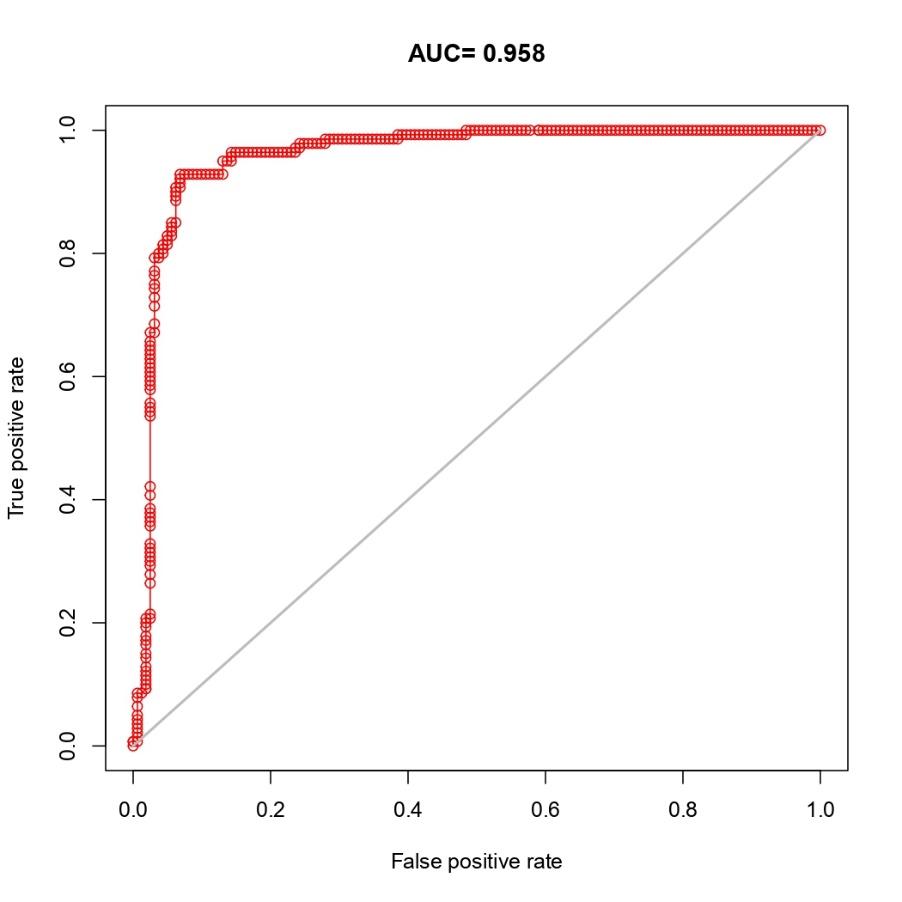


Figure S3. ROC curve of MaxEnt model.
